# Integrative Analysis of lncRNA-miRNA-mRNA Regulatory Network Reveals the Key lncRNAs Implicated Potentially in the Differentiation of Adipocyte in Goats

**DOI:** 10.3389/fphys.2022.900179

**Published:** 2022-05-05

**Authors:** Changsheng He, Yong Wang, Jiangjiang Zhu, Yanyan Li, Juan Chen, Yaqiu Lin

**Affiliations:** ^1^ Key Laboratory of Qinghai-Tibetan Plateau Animal Genetic Resource Reservation and Utilization of Education Ministry, Southwest Minzu University, Chengdu, China; ^2^ Key Laboratory of Qinghai-Tibetan Plateau Animal Genetic Resource Reservation and Exploitation of Sichuan Province, Southwest Minzu University, Chengdu, China; ^3^ College of Animal and Veterinary Science, Southwest Minzu University, Chengdu, China; ^4^ College of Food Science and Technology, Southwest Minzu University, Chengdu, China

**Keywords:** RNA-Seq, intramuscular adipocyte, subcutaneous adipocyte, goat, lncRNA, mRNA

## Abstract

Goats are popular in China because of their superior meat quality, delicate flesh, and unique flavor. Long noncoding RNAs (lncRNAs) play important roles in transcriptional and post-transcriptional regulation of gene expression. However, the effects of lncRNAs on adipocyte differentiation in goat has not been fully elucidated yet. In this investigation, we performed RNA-Seq analysis of intramuscular and subcutaneous adipocytes from Jianzhou Daer goat before and after differentiation, including both intramuscular preadipocytes (IMPA) vs. intramuscular adipocytes (IMA) and subcutaneous preadipocytes (SPA) vs. subcutaneous adipocytes (SA). A total of 289.49 G clean reads and 12,519 lncRNAs were obtained from 20 samples. In total, 3,733 differentially expressed RNAs (182 lncRNAs and 3,551 mRNAs) were identified by pairwise comparison. There were 135 differentially expressed lncRNAs (DELs) specific to intramuscular adipocytes, 39 DELs specific to subcutaneous adipocytes, and 8 DELs common to both adipocytes in these 182 DELs. Some well-known and novel pathways associated with preadipocyte differentiation were identified: fat acid metabolism, TGF-beta signaling pathway and PI3K-Akt signaling pathway. By integrating miRNA-seq data from another study, we also identified hub miRNAs in both types of fat cells. Our analysis revealed the unique and common lncRNA-miRNA-mRNA networks of two kinds of adipocytes. Several lncRNAs that regulate potentially goat preadipocyte differentiation were identified, such as XR_001918 647.1, XR_001917728.1, XR_001297263.2 and LNC_004191. Furthermore, our findings from the present study may contribute to a better understanding of the molecular mechanisms underlying in goat meat quality and provide a theoretical basis for further goat molecular breeding.

## Introduction

Fat is generally divided into subcutaneous fat, intermuscular fat and intramuscular fat. The amount of intermuscular fat is small and its composition is similar to subcutaneous fat, so it is generally considered that fat is mainly divided into subcutaneous fat and intramuscular fat (Timón et al., 2001). The distribution of fat in meat animals and the fat content of each part have an important impact on meat production and quality. Subcutaneous fat determines the carcass lean rate to a certain extent. The thicker the fat means the lower the lean meat rate. Intramuscular fat has an important influence on the flavor and tenderness of goat meat. Within a certain range, the quality of meat gradually improves with the increase of intramuscular fat content. One of the current research directions for meat is to study how to regulate the content of intramuscular fat and subcutaneous fat to improve meat quality of goats.

Adipogenesis is a complex process regulated by various transcription factors, non-coding RNA and signal pathways (Mattar et al., 2018). RNA sequencing (RNA-seq) is a revolutionary tool to identify differentially expressed genes (DEGs) regulating various biological processes. It enables us to discover new genes and therefore to describe unannotated transcriptional activity by identifying numerous noncoding transcripts (Wang et al., 2016). Long non-coding RNAs (lncRNAs) are non-coding RNAs with a length greater than 200 nucleotides and low protein coding ability. MicroRNAs (miRNAs) are a type of endogenous non-coding RNA with a length of approximately 22 nucleotides. Previous studies have shown that lncRNAs and mRNAs containing the same miRNA binding site can regulate mutual expression levels by competitively binding miRNAs. The recent explosion in knowledge demonstrating the importance of miRNAs and lncRNAs in the regulation of multiple major biological processes, which impacts the development, differentiation, and metabolism have brought these neglected molecular players (Huang et al., 2011; Wang and Chang, 2011; Ren et al., 2016). Some studies have shown that the adipogenic differentiation ability of intramuscular adipocytes is significantly lower than that of subcutaneous adipocytes. The expression of related genes is low, indicating that there are specific regulatory mechanisms for intramuscular fat and subcutaneous fat in animals (Zhou et al., 2010; Sun et al., 2013). In mammals, the differentiation of preadipocytes has been well studied, especially in bovine and porcine (Chen et al., 2016; Dong et al., 2016; Sun et al., 2016; Yonekura et al., 2016; Zappaterra et al., 2016). Recently, goat meat is gradually welcomed by consumers because of its high protein content, low fat and cholesterol content. Moreover, the consumers demand for goat meat and quality requirements continue to rise (Ivanovic et al., 2016; Teixeira et al., 2019). However, there are few studies on the molecular mechanism of the difference in the deposition of different fat tissues in goats.

Herein, we provided a comprehensive transcriptome profile on intramuscular and subcutaneous adipocytes in before and after differentiation of Jianzhou Daer goat. The results revealed the expression patterns of mRNAs and lncRNAs, which were important in the developmental stages of two different adipocytes. An integrated analysis of differentially expressed lncRNAs (DELs) and mRNAs (DEGs) was performed, and the lncRNA and its target miRNA/mRNA that could potentially regulate the differentiation of intramuscular and subcutaneous preadipocytes were screened through bioinformatics analysis. At the same time, a lncRNA-miRNA-mRNA interaction network was constructed based on miRNAs known to play a role in adipogenesis, which were identified by another study (manuscript in preparation). The lncRNA that may be combined with miRNA and mRNA was identified and verified by RT-qPCR technique. The results from the present investigation provided a theoretical basis for in-depth analysis of the regulation mechanism of goat fat cell differentiation and improvement of meat quality in goats.

## Materials and Methods

### Experimental Animals and Sample Collection

All experimental procedures were reviewed and approved by the Institutional Animal Care and Use Committee, Southwest Minzu University. Also, all the experiments complied with the requirements of the directory of the Ethical Treatment of Experimental Animals of China.

The 7-day-old male Jianzhou Daer goat (*Capra hircus*) (*n* = 5) was purchased from Sichuan Jianyang Dageda Aminal Husbandry Co., Ltd (Sichuan, China), being euthanized by bloodletting. The longissimus dorsi and subcutaneous fat were excised from the goats and minced. The isolation and culture of goat intramuscular and subcutaneous preadipocytes was performed as described assay by (Xu et al., 2018a). The intramuscular and subcutaneous adipocytes were isolated by using twice the volume of Type I collagenase (Sigma, St. Louis, MO, United States) in PBS (Hyclone, Logan, UT, United States). When the cell confluence reaches 80%, we discarded the medium (DMEM/F12 (Hyclone, Logan, UT, United States) with 10% fetal bovine serum (FBS) (Hyclone, Logan, UT, United States)) and added appropriate amount of trypsin (Hyclone, Logan, UT, United States), and the cells were digested at 37°C for 1 min. And then, the cells were harvested in a centrifuge tube, followed by a centrifuge at 800 rpm/min for 3 min to obtain cell pellet. The collected cells were re-suspended for subsequent experiments. The cells of passage 3, were used for experimental treatment. The cells were coaxed into differentiating using oleic acid induction solution (DMEM/F12 (Hyclone, Logan, UT, United States) containing 10% FBS (Hyclone, Logan, UT, United States) and 50 μm/L oleic acid (Sigma, St. Louis, MO, United States), and harvested after induction for 0 and 3 days (Xiong et al., 2018; Song et al., 2020; Xiong et al., 2021; Li et al., 2022). These cell samples were named intramuscular preadipocytes (IMPA), subcutaneous preadipocytes (SPA), intramuscular adipocytes (IMA) and subcutaneous adipocytes (SA), respectively, and five biological replicates were set for each group.

### Total RNA Extraction and Sequencing

A total of 20 cell samples were successfully collected. Total RNA was extracted using Trizol reagent (Takara, Dalian, China). RNA quality was determined using NanoPhotometer spectrophotometer and Agilent 2,100 bioanalyzer, which analyzes the integrity of the RNA （RIN）. The lowest RIN accepted for RNA analyses is 6.8. The lncRNA library is constructed using a chain-specific library. The method for synthesizing the first strand of cDNA by reverse transcription is the same as the normal method of NEB library construction. The difference is that when the second strand is synthesized, dTTP in dNTPs is replaced by dUTP. After that, cDNA end repair, A-tailing, ligation of sequencing adapters, and length screening were also performed, and then the second strand of cDNA containing U was degraded by USER enzyme, and then PCR amplification was performed to obtain a library. Finally, twenty libraries were sequenced at Novogene Co. Ltd. (Beijing, China) on Illumina HiSeq Sequencing System. The RNA-Seq dataset supporting the conclusions of this article is available in the Gene Expression Omnibus (GEO) at the National Center for Biotechnology Information (NCBI) under accession numbers GSE186988.

### Quality Control and Transcript Assembly

After sequencing, the raw data were stored in fastq format (de Sena Brandine and Smith, 2019). We removed reads containing connectors, poly-N and low quality from Raw Reads to get clean reads. The Q20, Q30 and GC content of the clean data were calculated (Bai et al., 2015). The obtained clean, high-quality data were used for further analyses. The clean, paired-end reads were aligned to the goat genome sequence assembly using HISAT2 (v2.0.4) (Kim et al., 2015), and the transcripts were assembled using both Scripture (beta2) (Guttman et al., 2010) and Cufflinks (v2.1.1) (Trapnell et al., 2010).

### Identification of lncRNAs and Their Expression Analysis

To ensure the quality of the obtained lncRNAs, three criteria were used to identify the desired lncRNAs in the transcriptome assemblies: 1) transcripts with length >200 bp and exon number ≥2 were selected; 2) Cuffcompare (v2.1.1) was used to calculate the read coverage of every transcript, and transcripts with an FPKM value (Cuffquant) of more than 0.05 were removed; and 3) the coding potential of the transcripts was predicted using the coding potential calculator (CPC <1) (Kong et al., 2007), Coding Noncoding Index (CNCI, v2) (Sun et al., 2013), and Pfam (v1.3) (Finn et al., 2016) protein domain families to further remove coding genes. Subsequent steps were performed based on the intersection of the results obtained using the twenty databases.

The FPKMs values of lncRNAs and mRNAs in IMPA, IMA, SPA and SA libraries were calculated by StringTie (v1.3.1), Ballgown (Pertea et al., 2016) and Cuffdiff (v2.1.1) (Trapnell C et al., 2010). The transcripts in the 20 libraries were analyzed for differential expression, and the *p* value was used to screen the DELs and DEGs between two different adipocyte samples. When *p* < 0.05, the lncRNAs and mRNAs are considered to be differentially expressed.

### Gene Functional Annotation

The trans function describes the co-expression relationship between lncRNAs and mRNAs. Pearson correlation coefficient (R > 0.95 or R < 0.95) was calculated by custom scripts. David was used to cluster the target genes among 20 samples for functional enrichment analysis of lncRNA target genes (Huang et al., 2009). Adjusted *p*-value < 0.05 was set as significant threshold. Go (gene ontology) can enrich and analyze the target genes of DELs. Goseq (Release2.12) (Young et al., 2010) is used to enrich and analyze the target genes of DELs. Adjusted *p* < 0.05 is considered to be the significant enrichment of GEGs. KEGG is a database (http://www.genome.jp/kegg/) for understanding the advanced functions and utilities of biological systems such as cells, organisms, and ecosystems (Kanehisa and Goto, 2000; Kanehisa et al., 2008; Kanehisa, 2019; Kanehisa et al., 2021). We used KOBAS (v2.0) (Mao et al., 2005) software to detect the pathway enrichment analysis of DELs target genes in the KEGG pathway.

### Differentially Expressed miRNA and Construction of lncRNA-miRNA-mRNA Networks

We have previously established small RNA libraries by RNA-Seq before and after differentiation of intramuscular and subcutaneous adipocytes (data not yet published). The raw reads were filtered to remove low quality reads and reads with connectors to obtain clean reads. Novoaligen software was used to match the clean reads with the miRBase database to identify known miRNAs, and MirDeep software (Xie et al., 2012) was used to predict novel miRNAs were finally quantified using Novoaligen and Samtools software and normalised using Reads Per Million (RPM). DEGSeq software (Wang et al., 2010) was used for DEMs analysis, and P adjust values were used to indicate the significance of differentially expressed genes. The edgeR software (Robinson et al., 2010) was used to calculate DEMs, in two adipocytes. The screening criterion for significant differences is *p*-value <0.05. miRWalk, miRanda, RNAhybrid, and Targetscan were used for screening DEMs target DELs and DEGs. Then, the intersections of co-expressed lncRNAs, miRNAs, and mRNAs in the four programs were used to construct a lncRNA–miRNA–mRNA network.

DELs and DEGs were combined with the target lncRNAs and mRNAs of DEMs respectively to obtain the miRNA-mRNA relationship pair and miRNA-lnRNA relationship pair. Finally, the lncRNA–miRNA–mRNA network was visualized using Cytoscape v3.7 software (Shannon et al., 2003).

### Validation of Gene Expression of by RT-qPCR Technique

Primers were designed using Primer-BLAST on the NCBI website (Table 1). 1 μg of RNA was synthesized into the first cDNA strand using the RevertAid First Strand cDNA Synthesis Kit (Thermo, Waltham, MA, United States), in accordance with the user manual. Ubiquitously expressed transcript gene (*UXT*) was used as a housekeeping gene (Xu et al., 2018b). The qPCR reaction procedure was composed of four steps, including pre-degeneration (95°C, 3 min), degeneration (95°C, 10 s), annealing (60°C, 10 s) and extension (72°C, 15 s), of which degeneration, annealing and extension were running for 40 cycles. Melt curve stage was 65–95°C in 0.5°C increments for 5s. Quantification of selected gene expression was performed using the comparative threshold cycle (2^−ΔΔCT^) method (Livak and Schmittgen, 2001). The experiment was repeated for three times.

**TABLE 1:** Primer sequences of lncRNA amplified by qRT-PCR.

Gene Name	Primer Sequence	Annealing (°C)	Amplicon size (bp)	Efficiency amplification (%)
XR_001917557.1	TCC​ATT​TTG​CCG​CAG​TGT​TC	60	167	99
	GAA​ATG​CAC​ACG​GCA​GAG​AC			
XR_001918647.1	AGC​TTG​GGA​GAT​GCA​CAA​AA	60	87	108
	GCC​AGC​ATA​TTG​GAC​ACC​CTT			
XR_001917728.1	CTC​TGT​GGG​CGA​TGA​CGA​AG	60	153	105
	TCT​CCA​TCA​CAC​CGG​ACC​AT			
LNC_004191	AGG​AAT​GGA​AAG​TGA​ACC​AGG​G	60	80	108
	AGC​TGT​GTT​CCT​CCC​CTA​CC			
XR_001295810.1	AAG​CAA​ACG​GTG​TCT​GGG​G	60	81	107
	AGA​GCA​ATG​GTC​AGC​TTG​GA			
XR_001917637.1	TGG​GCA​AGT​GAG​GGT​CTC​C	60	80	103
	CCC​TAC​AAG​CCT​CTT​CTC​CAT​C			
XR_001297263.2	CAC​AGG​GTG​AAT​GAC​TTG​GG	60	166	99
	AAT​AGG​GTG​CTT​GCA​GGT​AGG			
UXT	GCA​AGT​GGA​TTT​GGG​CTG​TAA​C	60	180	103
	ATG​GAG​TCC​TTG​GTG​AGG​TTG​T			

## Results

### Transcript Sequencing and Assembly

**FIGURE 1:**
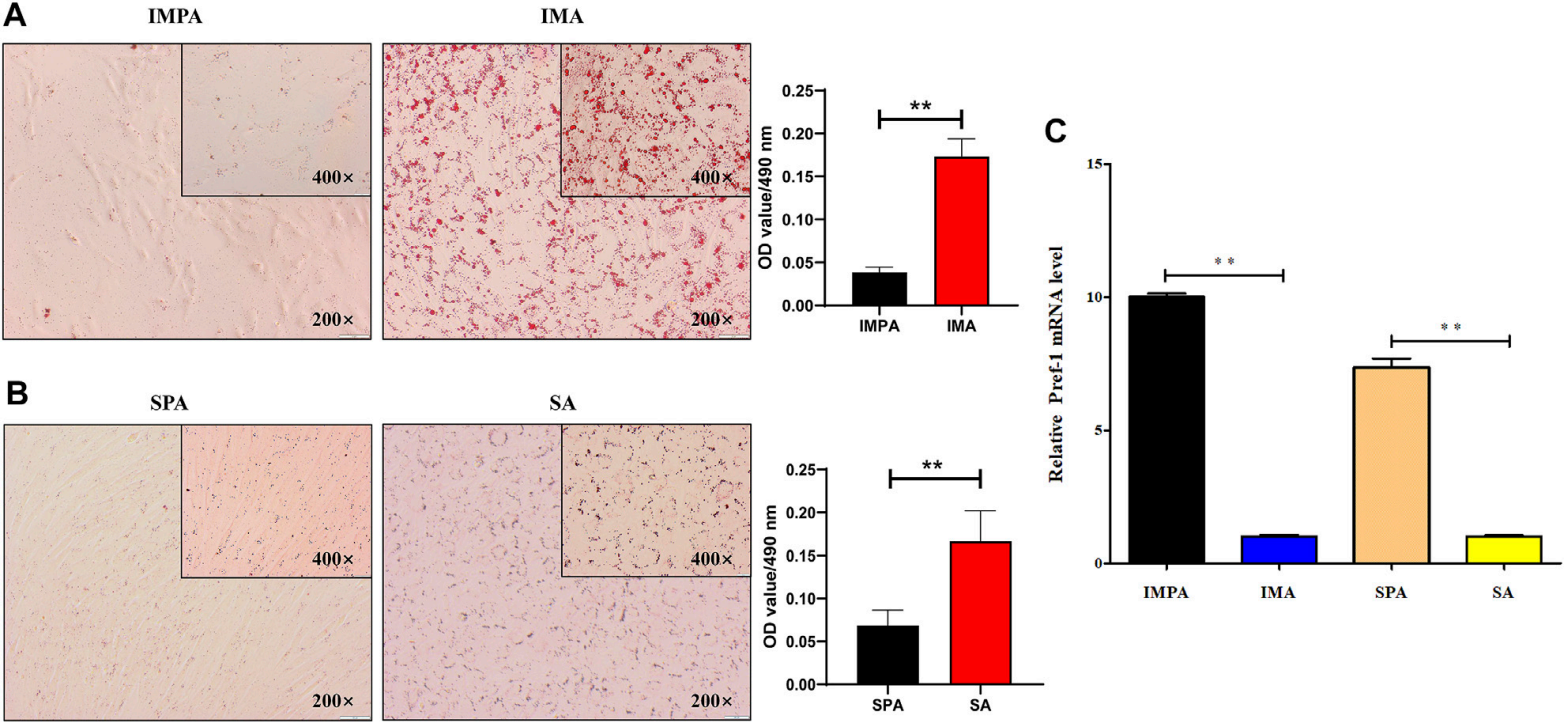
Adipocytes differentiated for 3 days **(A)** Oil-red O staining analysis in goat intramuscular adipocyte adipogenesis. “*”means *p* < 0.05 and “**” means *p* < 0.01, same below. **(B)** Oil-red O staining analysis in goat subcutaneous adipocyte adipogenesis. **(C)** The mRNA level of Pref-1 in goat intramuscular and subcutaneous adipocyte adipogenesis.

Three days after differentiation of intramuscular and subcutaneous adipogenesis, lipid droplets could be observed with Oil Red O staining (Figures 1A,B) and the relative expression level of the adipocyte differentiation marker gene Pref-1 was significantly lower than that of preadipocytes (Figure 1C), which could indicate that differentiated intramuscular and subcutaneous adipocyte models were successfully established. We established twenty cDNA libraries that represented two different adipocytes: intramuscular preadipocytes (IMPA, 1–5) and intramuscular adipocytes (IMA, 1–5) from the longissimus dorsi of 7-day-old Jianzhou Daer goat, subcutaneous preadipocytes (SPA, 1–5) and subcutaneous adipocytes (SA, 1–5) from the subcutaneous fat of 7-day-old Jianzhou Daer goat. The RNA sequencing obtained a total of 289.49 Gb of data, with each stage averaging 14.4745 Gb of data. The Q30 results in each sample were >91%, and the GC percentage was less than 52%, as listed in Table 2. More than 47.76% of the clean reads were perfectly mapped to the goat reference genome (assembly ARS1, https://www.ncbi.nlm.nih.gov/genome/?term = goat), and 44.56–87.33% uniquely mapped reads were obtained from the total mapped reads from the twenty samples (Table 3).

**TABLE 2:** Output statistics of the sequencing reads for each sample.

Sample Name	Raw Reads	Clean Reads	Clean Bases (G)	Error rate (%)	Q20 (%)	Q30 (%)	GC content (%)
IMA1	106965744	104026824	15.6	0.01	97.43	93.49	46.19
IMA2	97739340	95783582	14.37	0.01	97.38	93.41	45.44
IMA3	100355022	98296238	14.74	0.02	97.28	93.19	46.19
IMA4	87081698	85127438	12.77	0.02	97.30	93.26	45.62
IMA5	93900560	92052240	13.81	0.01	97.33	93.31	45.94
IMPA1	89914112	87780640	13.17	0.02	96.88	92.26	50.48
IMPA2	89869988	87544572	13.13	0.02	96.76	92.01	49.14
IMPA3	86513066	84676776	12.7	0.02	96.89	92.26	51.41
IMPA4	93072522	91173148	13.68	0.01	97.45	93.47	49.64
IMPA5	103727284	1,00918664	15.14	0.02	96.27	91.00	51.62
SA1	102257046	96016600	14.4	0.01	97.19	93.12	45.12
SA2	113958516	107224614	16.08	0.01	97.21	93.11	45.29
SA3	96569252	90603612	13.59	0.02	97.01	92.44	44.74
SA4	92196036	86251102	12.94	0.02	97.24	92.92	44.74
SA5	8,9102014	86443762	12.97	0.02	96.54	91.61	45.44
SPA1	89233018	87343234	13.1	0.02	97.03	92.56	45.46
SPA2	112263802	105239820	15.79	0.02	97.12	92.97	45.32
SPA3	119992036	112810026	16.92	0.02	97.08	92.87	45.82
SPA4	116537456	109357518	16.4	0.02	97.18	93.04	45.78
SPA5	128978020	121273002	18.19	0.02	97.15	93.00	45.75

**TABLE 3:** Summary of the clean reads alignment to the goat reference genome.

Sample Name	Total Reads	Total Mapped	Multiple Mapped	Uniquely Mapped
IMA1	104026824	58826296 (56.55%)	5007777 (4.81%)	53818519 (51.74%)
IMA2	95783582	52467109 (54.78%)	3378217 (3.53%)	49088892 (51.25%)
IMA3	98296238	64127686 (65.24%)	4344218 (4.42%)	59783468 (60.82%)
IMA4	85127438	49359285 (57.98%)	3294364 (3.87%)	46064921 (54.11%)
IMA5	92052240	53774996 (58.42%)	3822996 (4.15%)	4,9952000 (54.26%)
IMPA1	87780640	80670979 (91.9%)	6751338 (7.69%)	73919641 (84.21%)
IMPA2	87544572	70588932 (80.63%)	6111853 (6.98%)	64477079 (73.65%)
IMPA3	84676776	79535538 (93.93%)	7181504 (8.48%)	72354034 (85.45%)
IMPA4	91173148	78490007 (86.09%)	6695561 (7.34%)	71794446 (78.75%)
IMPA5	1,00918664	96226399 (95.35%)	8091611 (8.02%)	88134788 (87.33%)
SA1	96016600	47294011 (49.26%)	3673053 (3.83%)	43620958 (45.43%)
SA2	107224614	56076878 (52.3%)	4502794 (4.2%)	51574084 (48.1%)
SA3	90603612	45139764 (49.82%)	3788336 (4.18%)	41351428 (45.64%)
SA4	86251102	41189983 (47.76%)	2755466 (3.19%)	38434517 (44.56%)
SA5	86443762	42725042 (49.43%)	3277966 (3.79%)	39447076 (45.63%)
SPA1	87343234	49526419 (56.7%)	3284941 (3.76%)	46241478 (52.94%)
SPA2	105239820	57264925 (54.41%)	3543746 (3.37%)	53721179 (51.05%)
SPA3	112810026	63555694 (56.34%)	4360117 (3.87%)	59195577 (52.47%)
SPA4	109357518	59092897 (54.04%)	3924824 (3.59%)	55168073 (50.45%)
SPA5	121273002	64549280 (53.23%)	4580894 (3.78%)	59968386 (49.45%)

**FIGURE 2:**
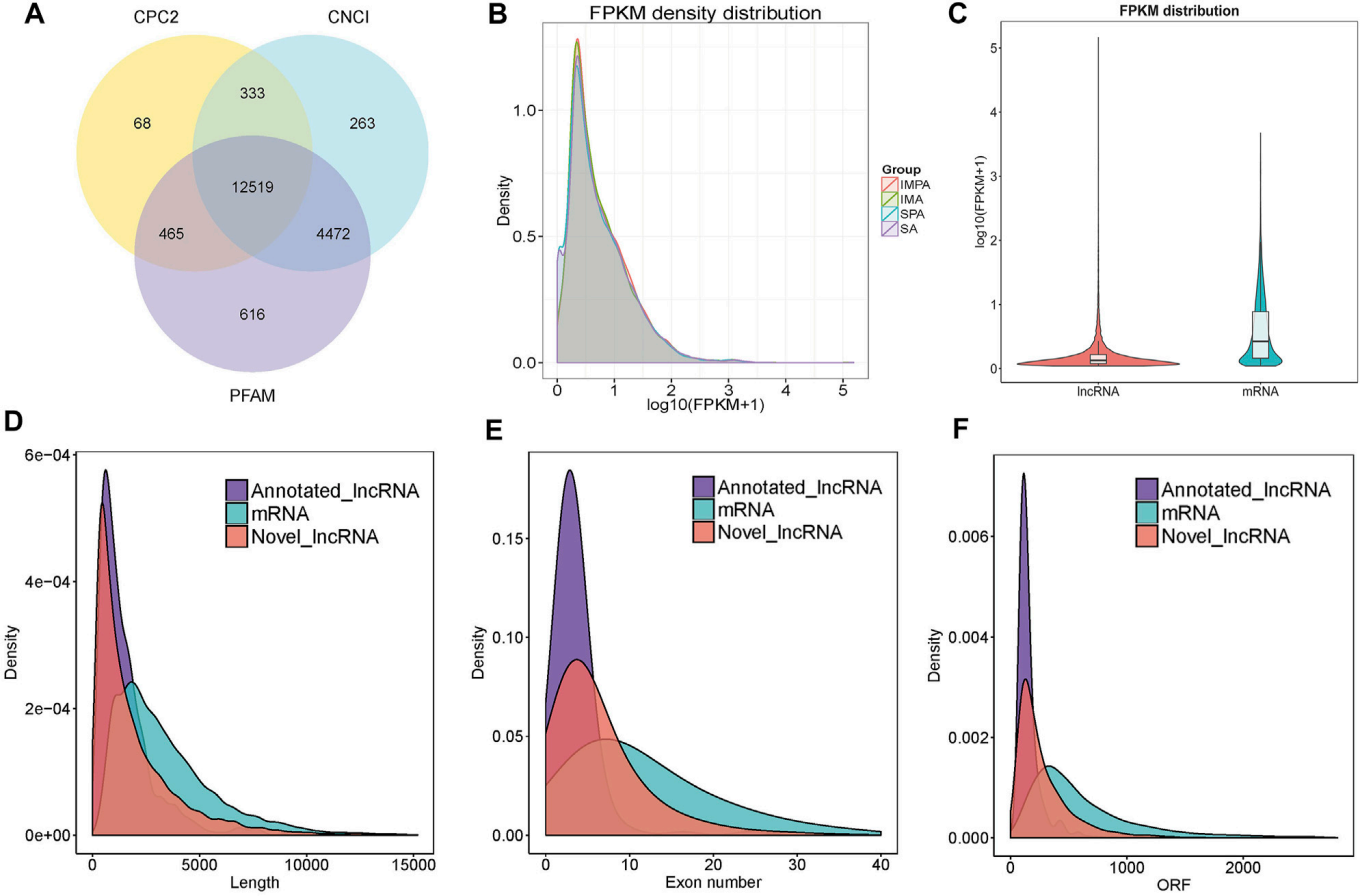
Identification and characterization of lncRNAs in goat, *Capra hircus*. **(A)** Venn diagrams of coding potential analysis by using stringent criteria. Three tools (CPC2, CNCI and PFAM) were employed to analyze the coding potential of lncRNAs. Those simultaneously shared by three analytical tools were designated as candidate lncRNAs and used in subsequent analysis. **(B)** Volcanic maps of differentially expressed RNA. The expression levels of mRNAs and lncRNAs were indicated by log10 (FPKM +1). **(C)** LncRNA, mRNA expression violin graph. The horizontal coordinate is the molecular type, the vertical coordinate is log10 (FPKM+1) and the width of each violin plot reflects the number of points at that expression level. **(D)** Length distribution of mRNAs and lncRNAs, unit of the length is bp. **(E)** Distribution of exon number in the mRNAs and lncRNAs. **(F)** Open reading frame (ORF) length distribution for mRNAs and lncRNAs.

Finally, 12519 assumed non-coding transcripts were retained by CNCI, CPC2 and PFAM softwares (Figure 2A), including 16.9% lncRNAs, 76.1% intronic lncRNAs, and 7% anti-sense lncRNAs. In addition, 43767 mRNAs were identified. The expression level of each RNA was standardized to fragments per kilobase of exon model per million mapped reads (FPKM), and it was found that a small part of them was not expressed or was expressed at a relatively low level. The expression levels of a large number of RNAs were mainly between 0.1 and 2 of log10 (FPKM+1), and a small number of genes had very high expression level (log10 |FPKM+ 1| > 2) (Figure 2B). LncRNAs are lesser expressed than mRNAs (Figure 2C), the extremely significant differences of genes were helpful to explore the molecular mechanism of fat deposition in different tissues.

### Characteristics of lncRNAs and mRNAs in Intramuscular and Subcutaneous Adipocytes of Goats Before and After Differentiation

Since most lncRNAs are produced by RNA polymerase II transcription, they have structural features similar to those of mRNAs (Gao et al., 2017). Therefore, in order to observe the characteristics of lncRNAs and the difference between lncRNAs and mRNAs, we compared the length of lncRNAs and mRNAs, including the number of exons, and the open reading frame (ORF). The results showed that most of lncRNAs tended to be shorter in length, and they contained less exons than mRNAs (Figure 2D). Also, the length of ORFs in the lncRNAs was shorter than those of the mRNAs (Figures 2E,F).

### Differentially Expressed lncRNAs and mRNAs in Goat Different Adipocytes

**FIGURE 3:**
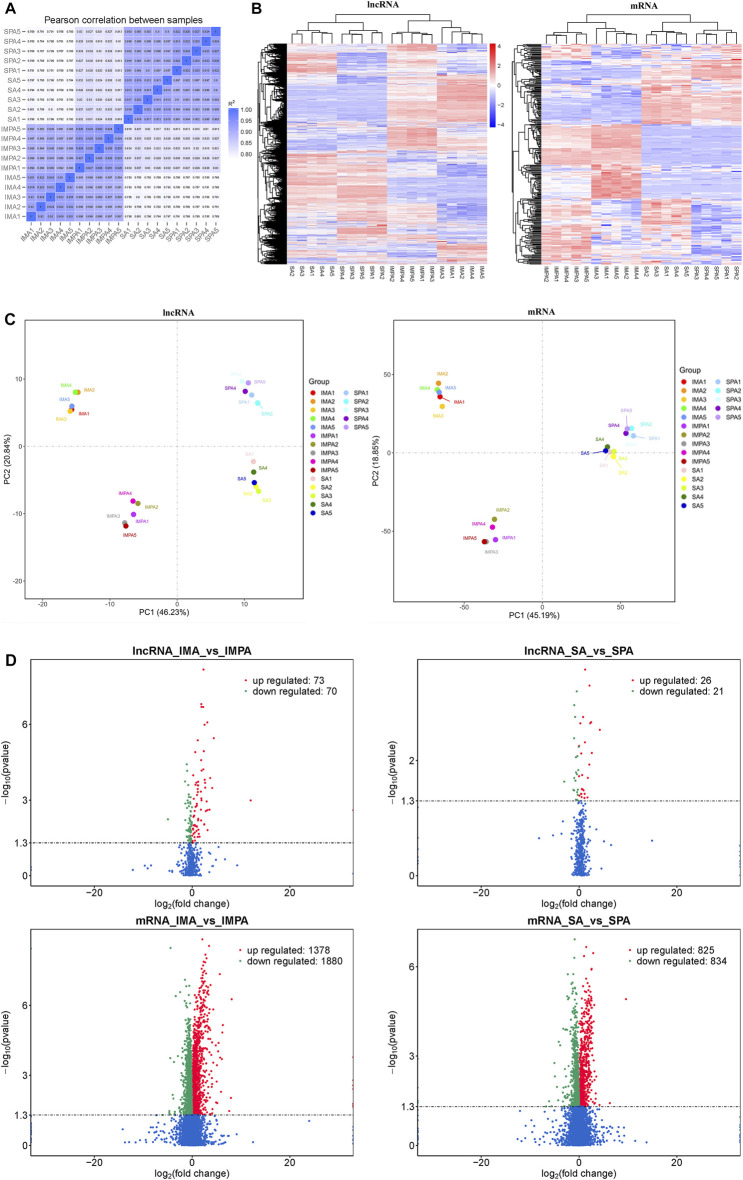
Differentially expressed coding genes and lncRNAs in goat adipocytes between intramuscular and subcutaneous of differentiation (Fold Change ≥2 and P-adjust value ≤ 0.05). **(A)** Representative of the degree of similarity between samples. The correlation coefficient is represented by color; deeper color represents a stronger correlation. **(B)** Clustering analysis of differentially expressed lncRNAs and mRNAs in IMA vs. IMPA and SA vs. SPA. Red means highly expressed genes and blue means low expressed genes. The color is from red to blue, which means log10(FPKM+1) from large to small. **(C)** PCA plots of differentially expressed lncRNAs and mRNAs in IMA vs. IMPA and SA vs. SPA. Scatter plots are drawn using principal component 1 and principal component 2 as the x- and *y*-axes.**(D)** Volcano plot of differentially expressed lncRNAs and mRNAs in IMA vs. IMPA and SA vs. SPA. Green and red represent downregulated and upregulated expression, respectively.

The correlation coefficient can represent the degree of similarity between samples. We found that the data between SPA and SA, and between IMPA and IMA were highly correlated in expression (Figure 3A). We found 143 lncRNAs and 33258 mRNAs differentially expressed between IMA and IMPA (73 lncRNAs and 1,378 mRNAs were up-regulated, 70 lncRNAs and 1880 mRNAs were down-regulated), 47 lncRNAs and 1,659 mRNAs differentially expressed between SA and SPA (26 lncRNAs and 825 mRNAs were up-regulated, 21 lncRNAs and 834 mRNAs were down-regulated) (Figure 3B) (Supplements 1, 2, 5, 6). The PCA score plots for each of mRNA and lncRNA showed a high degree of similarity in the same group of samples (Figure 3C). To further explore the differences in lncRNAs expression between subcutaneous and intramuscular adipocytes at different developmental stages, we performed a clustered heat map on the differentially expressed genes. The results of the cluster analysis showed that distinct lncRNAs expression patterns are associated with the differentiation of both subcutaneous and intramuscular adipocytes in goats (Figure 3D).

### Function Prediction of lncRNAs and Corresponding Genes During Intramuscular and Subcutaneous Preadipocyte Differentiation

LncRNAs not only can regulate the expression of neighboring protein-coding genes through a cis mechanism (Bu et al., 2012; Han et al., 2012), but also regulate the expression of genes located on other chromosomes via a trans mechanism (Yang et al., 2016; Cai et al., 2017). In this study, co-expression analysis was used to predict the potential target genes of DELs via a trans mechanism. The absolute value of Pearson’s correlation coefficient was set as greater than 0.95 (Supplement 7). There were 39012 lncRNA-mRNA relationship pairs. Among them, LNC_004374, LNC_007272, LNC_010037.

LNC_010037, LNC_004066, LNC_010143 and other lncRNAs can target genes related to adipocyte differentiation or lipid metabolism, CD36 (Lewis, 2006), FABP3 (Nakamura et al., 2013), FGF11 (Lee et al., 2019), FOXO6 (Lee et al., 2019), SMAD1 (Shin et al., 2016), TGFB2 (Takahashi et al., 2019), FGFR2 (Fischer et al., 2017). The results are shown in Table 4.

**TABLE 4:** Expression of some lncRNAs and their associated genes affecting adipogenesis.

Target Gene	Description	lncRNA
*CD*36	CD36 molecule	LNC_004374 LNC_007272 LNC_010037 LNC_004066 LNC_010143
		LNC_003287 LNC_000379 LNC_000462 LNC_005589 LNC_002362
		LNC_011316 LNC_011269 LNC_001565 LNC_006348 LNC_002032
		LNC_000679 LNC_002558 LNC_011971 LNC_009810 XR_001919420.1
*FABP*3	fatty acid binding protein 3	XR_001917639.1 LNC_010010 XR_309714.3 XR_001918356.1
		LNC_005049 LNC_008023 LNC_007295 XR_001919935.1
		XR_001918074.1 LNC_009465 XR_001917605.1 LNC_004888
*FGF*11	fibroblast growth factor 11	LNC_009670 LNC_011765 LNC_011764 LNC_010048
*FOX O* 6	forkhead box O6	XR_001917700.1 XR_001918450.1 XR_001917557.1 XR_001919481.1 XR_001919828.1 LNC_007210 LNC_007370 LNC_006351 LNC_007188 LNC_006638 LNC_009693 LNC_010010 LNC_011790 LNC_002248
		LNC_000560 XR_001917649.1 XR_001918167.1 LNC_006658
		LNC_008810
*SMAD*1	SMAD family member 1	LNC_009792 LNC_007731 LNC_000706 LNC_008467 LNC_006192
		LNC_004878
*TGFB*2	transforming growth factor beta 2	LNC_011039 XR_001917557.1 XR_001917639.1 LNC_007210
		LNC_007370 LNC_010010 LNC_005049 XR_001917649.1
		XR_001918074.1 LNC_000622 LNC_009465 XR_001917605.1
*FGFR*2	fibroblast growth factor receptor 2	LNC_004374 LNC_000260 LNC_007272 LNC_001963 LNC_006095
		LNC_004066 LNC_010140 LNC_001914 LNC_003262 LNC_006919
		LNC_007053 LNC_000379 LNC_006649 LNC_009821 LNC_003963
		LNC_002980 LNC_010402 LNC_011269 LNC_012328 LNC_000820
		LNC_006348 LNC_002032 LNC_009618 LNC_004423 LNC_002558
		LNC_009048 LNC_011971 LNC_009810 LNC_001111

**FIGURE 4:**
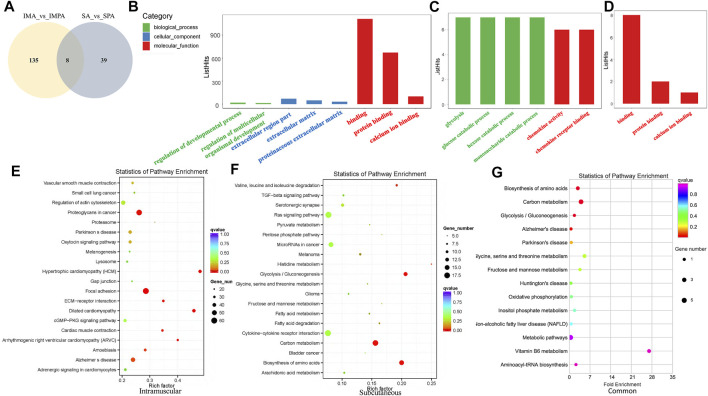
GO and KEGG enrichment analyses of differentially expressed lncRNAs in goat different adipocytes. **(A)** Venny maps of differentially expressed lncRNAs during intramuscular and subcutaneous preadipocyte differentiation. **(B)** Categories of biological processes, cellular components and molecular functions of the target genes of differentially expressed lncRNAs (intramuscular adipocytes). **(C)** Categories of the biological processes and molecular functions of the target genes of differentially expressed lncRNAs (subcutaneous adipocytes). **(D)** Categories of molecular functions of the target genes of differentially expressed lncRNAs (both subcutaneous and intramuscular adipocytes). **(E)** Scatter plot of the top 20 pathways enriched for differentially expressed lncRNAs in intramuscular adipocytes before and after differentiation. **(F)** Scatter plot of the top 20 pathways enriched for differentially expressed lncRNAs in subcutaneous adipocytes before and after differentiation. **(G)** Scatter plot of the top 15 pathways enriched for differentially expressed lncRNAs in both intramuscular and subcutaneous adipocytes before and after differentiation. The abscissa represents the richness factor, and the ordinate represents the enriched pathway terms. The Q-value represents the corrected *p* value.

We drew Venn diagrams (Figure 4A) to visualize the number of differential lncRNAs that are common versus unique to the two adipocyte differential differentiation processes. 135 DELs were specific to intramuscular adipocytes, 39 were specific to subcutaneous adipocytes, and 8 DELs were common to both adipocytes. Through the use of Goseq, we compare the GO classifications of DELs using mRNAs showing a correlated expression. (adjusted *p* < 0.05) (Young et al., 2010). The DELs target genes are enriched in biological process (BP), cellular component (CC) and molecular function (MF) (Table 5). Unique DELs target genes of goat intramuscular adipocytes before and after differentiation are mainly enriched in binding (Figure 4B), and the unique DELs target genes of subcutaneous adipocytes are mainly enriched in glucose metabolism and chemokine (Figure 4C), while the shared DELs target genes are mainly enriched in binding (Figure 4D).

**TABLE 5:** GO enrichment analysis of the target genes of IncRNAs in intramuscular and subcutaneous adipocytes.

	IMA VS. IMPA	SA VS. SPA	Common
biological process	30	28	0
cellular component	148	0	0
molecular function	1882	12	11

The KEGG annotation results of the differentially expressed target genes of lncRNAs are classified according to the pathway types of the KEGG database. As shown in Figure 4E, the unique DELs target genes of intramuscular adipocytes before and after differentiation are most significantly enriched in hypertrophic cardiomyopathy (HCM), dilated cardiomyopathy, focal adhesion, arrhythmogenic right ventricular cardiomyopathy (ARVC), ECM-receptor interaction, proteoglycans in cancer and cardiac muscle contraction (adjusted *p* < 0.05). In addition, the analyzed genes also was enriched in fat acid metabolism and TGF-beta signaling pathway, which are related to adipocyte formation (Supplement 10). The unique DELs target genes of subcutaneous adipocytes before and after differentiation are most significantly enriched in biosynthesis of amino acids, glycolysis/Gluconeogenesis, carbon metabolism, valine, leucine and isoleucine degradation, histidine metabolism, fatty acid degradation, melanoma, fatty acid metabolism (adjusted *p* < 0.05), among which fatty acid degradation, fatty acid metabolism, TGF-beta signaling pathway, PI3K-Akt signaling pathway have a significant impact on the adipogenesis (Figure 4F). The shared DELs target genes are most significantly enriched in biosynthesis of amino acids, carbon metabolism, glycolysis/gluconeogenesis and alzheimer’s disease (adjusted *p* < 0.05) (Figure 4G).

### Construction of Regulation Networks for Adipocytes in Goats

**FIGURE 5:**
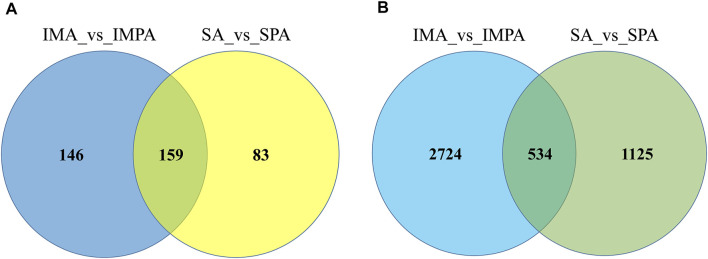
Venny maps of differentially expressed RNAs. Venny maps showing specific miRNAs **(A)** and mRNAs **(B)**, respectively, in intramuscular adipocytes or subcutaneous adipocytes.

As to miRNAs analysis, 210 known miRNAs and 95 novel miRNAs were found to have significantly different expression between intramuscular preadipocytes and adipocytes (Supplement 3), 175 known miRNAs and 67 novel miRNAs were found to have significantly different expression between subcutaneous preadipocytes and adipocytes (Supplement 4). Among them, we obtained 146 differentially expressed miRNAs (DEMs) unique to intramuscular adipocytes, 83 (DEMs) unique to subcutaneous adipocytes, and 159 (DEMs) shared by two adipocytes (Figure 5A). After targeted binding prediction, 30,6571 mRNAs (Supplement 9) and 32,029 lncRNAs.

**FIGURE 6:**
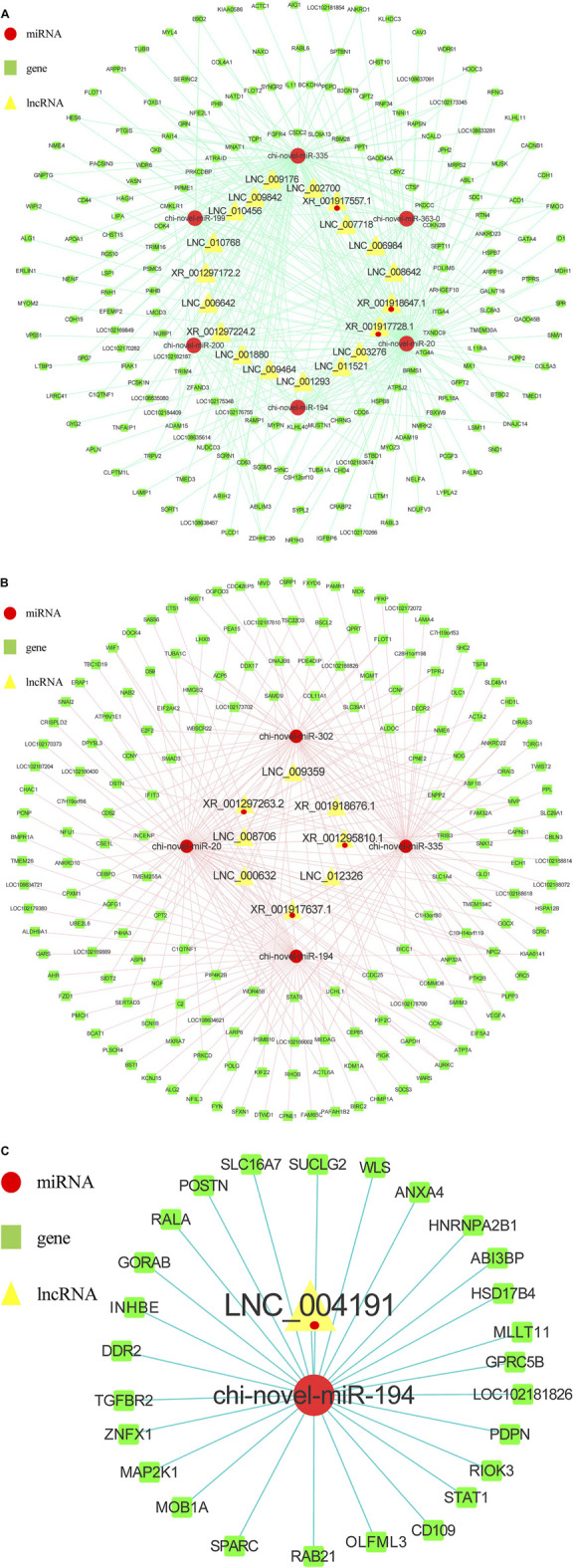
lncRNA–miRNA–mRNA network. **(A)** Regulatory network of intramuscular adipocytes before and after differentiation. Nodes with Closeness Centrality >6.4815E-4 are discarded. Closeness Centrality is the reciprocal of the sum of the shortest paths from a point to all other points, and the larger it is, the shorter the path from that point to all other points and the closer it is to the center in space. **(B)** Regulatory network of subcutaneous adipocytes before and after differentiation. **(C)** There is a common network of intramuscular and subcutaneous adipocytes before and after differentiation.

32,029 lncRNAs (Supplement 8) were found to have potential targeted binding relationships with DEMs. Combined with the sequenced intramuscular adipocyte-specific 2,724 DEGs and 135 DELs, 6,572 miRNA-mRNA relationship pairs and 1,240 miRNA-lncRNA relationship pairs were obtained (Figure 5B). The 1,125 DEGs and 39 DELs unique to subcutaneous adipocytes were combined to obtain 3,287 miRNA-mRNA relationship pairs and 307 miRNA-lncRNA relationship pairs (Figure 5B). At the same time, the intersection with the shared 534 DEGs and 8 DELs yielded 1,681 miRNA-mRNA relationship pairs and 52 miRNA-lncRNA relationship pairs. The lncRNA-miRNA-mRNA interaction network was constructed by Cytoscape software, and the top 20 miRNAs were selected after calculating the degree value of each factor (Table 6). Among them, we found miR-20, miR-194, miR-335, miR-363, miR-200, miR-199, and miR-302 related to fat formation, thereby, we constructed a ceRNA network view. As shown in Figure 6A, the unique lncRNA-miRNA-mRNA network of intramuscular adipocytes includes 30 lncRNAs, six miRNAs and 426 mRNAs, and the lncRNA-miRNA-mRNA network unique to subcutaneous adipocytes includes 8 lncRNAs, four miRNAs and 182 mRNAs (Figure 6B). Whereas, two types of adipocyte-common lncRNA-miRNA-mRNA networks include 1 lncRNA, 1 miRNAs and 26 mRNAs (Figure 6C).

**TABLE 6:** Node degree of mRNA-miRNA-lncRNA regualatory network.

Factor Type	Intramuscular Unique	Degree	Subcutaneous Unique	Degree	Common	Degree
miRNA	chi-novel-miR-174	172	chi-novel-miR-184	92	chi-novel-miR-302	35
miRNA	chi-novel-miR-29	151	chi-novel-miR-183	92	chi-novel-miR-29	34
miRNA	chi-novel-miR-69	126	chi-novel-miR-182	92	chi-novel-miR-69	31
miRNA	chi-novel-miR-68	126	chi-novel-miR-20	63	chi-novel-miR-68	31
miRNA	chi-novel-miR-67	126	chi-novel-miR-302	59	chi-novel-miR-67	31
miRNA	chi-novel-miR-407	115	chi-novel-miR-174	57	chi-novel-miR-174	28
miRNA	chi-novel-miR-207	115	chi-novel-miR-69	57	chi-novel-miR-194	27
miRNA	chi-novel-miR-364	115	chi-novel-miR-68	57	chi-novel-miR-193	27
miRNA	chi-novel-miR-362	114	chi-novel-miR-67	57	chi-novel-miR-51	25
miRNA	chi-novel-miR-20	113	chi-novel-miR-29	54	chi-novel-miR-50	25
miRNA	chi-novel-miR-308	111	chi-novel-miR-335	53	chi-novel-miR-49	25
miRNA	chi-novel-miR-307	111	chi-novel-miR-244	53	chi-novel-miR-48	25
miRNA	chi-novel-miR-306	111	chi-novel-miR-243	53	chi-novel-miR-20	24
miRNA	chi-novel-miR-194	108	chi-novel-miR-407	47	chi-novel-miR-185	24
miRNA	chi-novel-miR-193	108	chi-novel-miR-207	47	chi-novel-miR-184	24
miRNA	chi-novel-miR-335	106	chi-novel-miR-162	46	chi-novel-miR-183	24
miRNA	chi-novel-miR-363	106	chi-novel-miR-194	46	chi-novel-miR-182	24
miRNA	chi-novel-miR-200	94	chi-novel-miR-193	46	chi-novel-miR-4	23
miRNA	chi-novel-miR-199	89	chi-novel-miR-292	46	chi-novel-miR-335	21
miRNA	chi-novel-miR-334	88	chi-novel-miR-4	41	chi-novel-miR-384	20

### Verification of lncRNA Expression Profiles Using qRT-PCR

**FIGURE 7:**
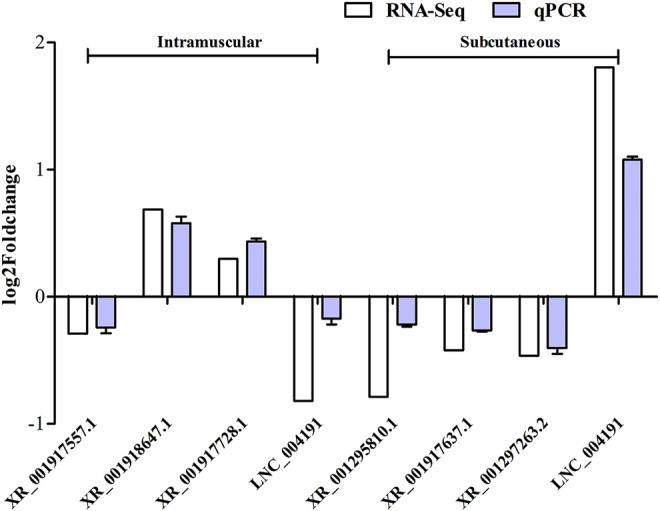
Validation of lncRNAs in intramuscular and subcutaneous adipocytes using RT-qPCR technique. Data were analyzed by the 2^−ΔΔCt^ method where the *UXT* was used as a reference gene. Each column represents the mean ± SE. White bars represent read from RNA-Seq, and blue bars represent the results from qRT-PCR analysis.

To verify the reliability of RNA-seq results, seven candidate lncRNAs were randomly selected from the DELs obtained from the screening, and *UXT* was used as an internal reference for qRT-PCR analysis. The results showed that XR_001917557.1, and LNC_004191 were significantly down-regulated in expression during the differentiation of intramuscular adipose tissue. Whereas, the XR_001918647.1, XR_001917728.1 were significantly up-regulated in expression during the differentiation of intramuscular adipose tissue. XR_001295810.1, XR_001917637.1 and XR_001297263.2 were significantly down-regulated expression during the differentiation of subcutaneous adipose tissue, and the LNC_004191 was significantly up-regulated expression during the differentiation of subcutaneous adipose tissue, These results were consistent with the trend of RNA-seq, indicating the credibility of the RNA-seq results (Figure 7).

## Discussion

The distribution and deposition of adipose tissue in different parts of the body are the key factors affecting carcass quality and meat flavor. Subcutaneous fat mainly affects carcass quality. Intramuscular fat (IMF) is the material basis of marbling, and an important factor affecting meat flavor. A large number of studies have shown that IMF is directly involved in the formation of meat tenderness, juiciness and flavor (Van Laack et al., 2001; Suzuki et al., 2005). Goat is an indispensable animal in China’s agricultural production, and the molecular regulation mechanism of its lipid deposition has not been fully elucidated yet.

LncRNA is a kind of noncoding RNA longer than 200 nt, which has attracted substantial attention in the last few years. Studies have shown that lncRNAs regulate metabolic tissue development and function, including adipogenesis, hepatic lipid metabolism, islet function, and energy balance (Alvarez-Dominguez et al., 2015; Chen et al., 2015; Luan et al., 2015; You et al., 2015; Zhao and Lin, 2015). Despite the fact that many studies have indicated the importance of lncRNAs in different tissues, little is known about their biological function in goat fat deposition, especially in the differentiation of goat intramuscular and subcutaneous preadipocytes. To the best of our knowledge, our study is the first to screen for lncRNAs and mRNAs regulating goat preadipocyte differentiation by sequencing and annotating the transcriptome of intramuscular and subcutaneous preadipocytes. A total of 1,118,110,544 reads were successfully mapped to the goat reference genome assembly. We identified 12,519 lncRNAs. The average sequence length of lncRNAs was shorter than that of mRNAs, and the number of exons was less than that of mRNAs, with the ORF length being shorter than that of mRNAs. Our results indicated that the predicted lncRNAs were shorter with fewer exons than mRNAs, which are in agreement with the those reproted in previous studies (Trapnell et al., 2010; Ran et al., 2016; Wang et al., 2016). The Pearson correlation (*R* ^2^) of each sample is greater than 0.8, which indicated that our experiment was reliable and the sample selection was reasonable.

LncRNA functions by regulating mRNA. At present, the mechanism of interaction between lncRNA and mRNA is not clear. We predict the biological function of lncRNAs through its co-expression with protein coding genes. Consequently, we found that many target genes of DELs were also differentially expressed in goat intramuscular and subcutaneous preadipocytes. This suggested that lncRNAs may function through complementary target genes, which can play critical roles in the differentiation of goat intramuscular and subcutaneous preadipocytes. For example, SMAD1 is a target gene of the differentially expressed lncRNAs LNC_009792, LNC_007731, LNC_000706, LNC_008467, LNC_006192 and LNC_004878, and it has been reported to regulate the differentiation of preadipocytes (Shin et al., 2016). These findings suggest that these lncRNAs might be involved in the differentiation of intramuscular preadipocytes by affecting the expression of SMAD1. However, there is no DEGs in the differentiation process of subcutaneous preadipocytes in our selected fat development related genes, so we speculate that the network regulating intramuscular adipogenesis is more complex than subcutaneous fat. The higher number of DELs, DEMs and DEGs in IMF than that of subcutaneous fat also supported this, which is consistent with the fact that intramuscular preadipocytes have stronger ability to deposit fat than that of subcutaneous preadipocytes (Chen et al., 2010).

To explore the similarities and differences of different adipocytes, DELs target genes (IMPA vs. IMA and SPA vs. SA) were subjected to GO and KEGG pathway enrichment analyses. We found that few common term was found between the IMPA vs. IMA and SPA vs. SA comparisons. Several pathways involved in preadipocyte differentiation were previously identified, including the TGF-β signaling pathway (IMF and subcutaneous fat) (Stewart et al., 2010), PI3K/AKT signaling pathway (subcutaneous fat) (Dong et al., 2016), and arachidonic acid metabolism (subcutaneous fat) (Nikolopoulou et al., 2014). For example, the LncRNA GAS5 inhibits lipogenesis in 3T3-L1 cells through the miR-21a-5p/PTEN signaling pathway (Liu et al., 2018). FDNCR1 affects porcine lipogenesis by competitively binding miR-204 to regulate the TGF-β pathway (Zhang et al., 2020). However, for some pathways identified here, their involvement in the goat preadipocyte differentiation process is being reported for the first time. Interestingly, in the pathway analysis, we found that the components of two pathways, fatty acid metabolism (IMF and subcutaneous fat) and fatty acid degradation (subcutaneous fat), which have been reported to be involved in lipid metabolism, and were enriched in the entire process of differentiation of intramuscular and subcutaneous preadipocytes (Wang et al., 2006; Jump, 2011). The common enrichment pathways during differentiation of both adipocytes involve amino acid metabolism, gluconeogenesis and carbohydrate metabolism, suggesting that IMF and subcutaneous fat are largely different in differentiation pathways and lipid metabolism pathways, while there are similarities in communication with other metabolic pathways. In addition to the specific pathways of the two adipocytes, there are also differences in the common components of the two pathways. Just as IMF is enriched in 19 target genes of TGF-β signaling pathway, 8 target genes of fatty acid metabolism, and subcutaneous fat is enriched in 8 and 7 targat genes. Here, we hypothesize that there are differences in the pathways regulating intramuscular and subcutaneous adipose differentiation, and that there are differences in the downstream target genes in the same pathways, which indirectly demonstrates that gene expression is tissue-specific in goats.

**FIGURE 8:**
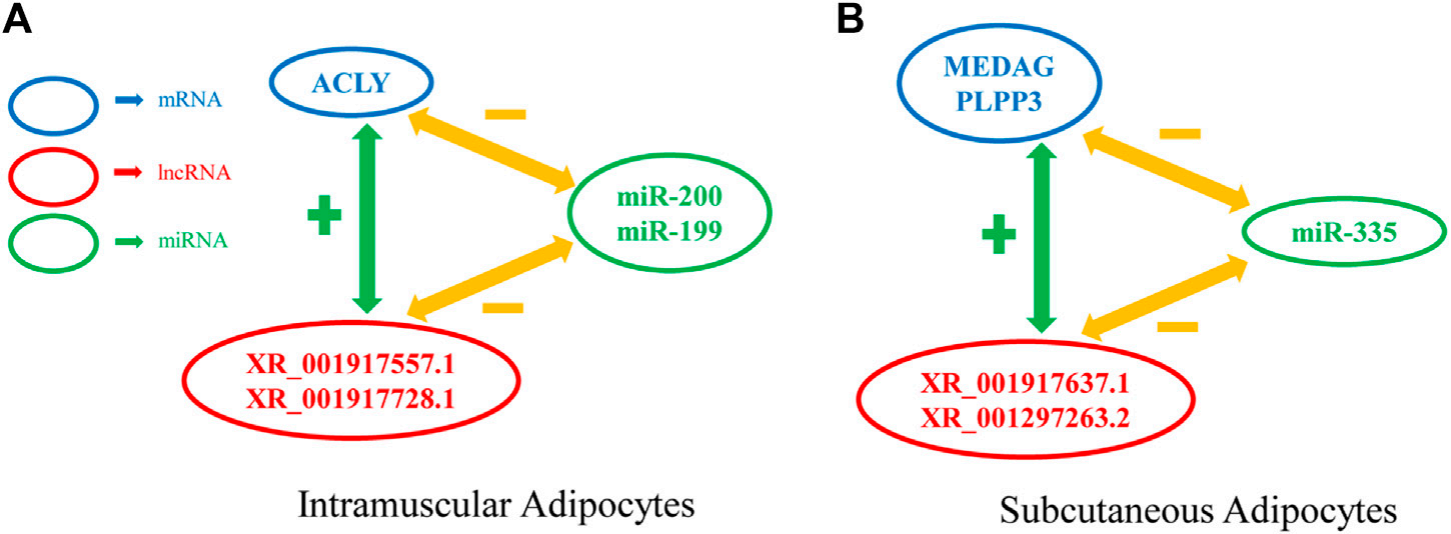
Example of a pure sponge prediction module. **(A)** A pure sponge prediction module extracted from intramuscular adipocytes network. **(B)** A pure sponge prediction module extracted from intramuscular adipocytes network.

To date, many genes have been reported to regulate the differentiation of preadipocytes. However, few studies have been conducted on the roles of lncRNAs in intramuscular and subcutaneous preadipocytes differentiation. The molecular and cellular mechanisms regulating goat preadipocytes differentiation are thus still poorly understood. Here, we constructed a miRNA-lncRNA-mRNA interaction network, and calculated the degree (the number of times each factor interacts with other factors) of each factor through Cytoscape (CentiScaPe (Scardoni et al., 2009)). We selected the top 20 miRNAs in the dgree, and found that miR-20 (Wang et al., 2008), miR-194 (Jeong et al., 2014), miR-335 (Fernandez-Hernando et al., 2011), miR-363 (Chen et al., 2014), miR-200 (Kennell et al., 2008), miR-199 (Shi et al., 2014), and miR-302 (Kim et al., 2014) were related to fat development, and visualized them in Cytoscape. We identified a number of highly connected lncRNAs and mRNAs in the three modules, including the two kinds of adipocytes are unique and common. For example, XM_005693834.3 (ACLY) (Han et al., 2016) and XM_013975359.2 (ANGPT2) (Bae et al., 2020) are unique to IMF, and XM_018056618.1 (MEDAG) (Li et al., 2021) and XM_005678357.3(PLPP3) (Bae et al., 2016) are unique to subcutaneous fat, whereas, XM_018040030.1 (CAPN10) (Patel and Lane, 1999) and XM_018050348.1 (TGFβ1) (Stewart et al., 2010) are shared by the two kinds of preadipocytes in differentiation processes. Seven DELs from the IMF vs. subcutaneous fat comparison were validated by qRT-PCR technique and the results were in excellent generally agreement with the RNA-seq findings. This suggests that our RNA-seq findings are reliable. Triangles miRNA-mRNA-lncRNA should be represented by a gene expression negatively correlated between miRNA-mRNA and miRNA-lncRNA and a positively correlated between lncRNA and mRNA as discussed in a pure sponge prediction module (Paci et al., 2014). Therefore, we constructed several examples of a pure sponge module based on qPCR-validated lncRNAs (Figure 8). Some notable phenomena such as: it is expected that both XR_001917637.1 and XR_001297263.2 should be upregulated in subcutaneous adipocytes to promote the expression of PLPP3 and MEDAG, yet both lncRNAs are actually downregulated in subcutaneous adipocytes. This phenomenon suggests that the triangle of miRNA-mRNA-lncRNA is a complex regulatory network, with negatively correlated gene expression between miRNA-lncRNAs or positively correlated gene expression between lncRNA-mRNAs contributing to this difference in final outcome. Our next experimental plan is to investigate the interactions between mRNA, lncRNA, and miRNA.

In conclusion, we first generated the expression profiles of lncRNAs from intramuscular and subcutaneous adipocytes of Jianzhou Daer goat (IMA vs. IMPA and SA vs. SPA) based on RNA-Seq technique. We found that the number of lncRNAs regulating IMF differentiation was more than that of subcutaneous adipocytes. Our results suggest that those lncRNAs might play important roles in adipocyte differentiation. Collectively, this study takes the first step toward understanding the molecular mechanisms underlying variations in goat adipogenesis. Also, our results provided a theoretical basis for molecular breeding to improve the meat quality in goats.

## Data Availability

The datasets presented in this study can be found in online repositories. The names of the repository/repositories and accession number(s) can be found below: https://www.ncbi.nlm.nih.gov/geo/query/acc.cgi?acc=GSE186988 GEO accession: GSE186988.
